# Plant-Mediated Synthesis of Silver Nanoparticles Using *Salvia tomentosa*: Characterization and Evaluation of Their Multifunctional Biological Activities, Including DNA Binding

**DOI:** 10.3390/nano16110679

**Published:** 2026-05-29

**Authors:** Havva Karahan, Ufuk Yildiz, Zeynep Şahintaş, Hatice Çölgeçen

**Affiliations:** 1 Department of Biology, Science Faculty, Zonguldak Bülent Ecevit University, Zonguldak 67100, Turkey; colgecen@beun.edu.tr; 2 Department of Chemistry, Science Faculty, Zonguldak Bülent Ecevit University, Zonguldak 67100, Turkey; ufukyildiz@beun.edu.tr (U.Y.); zeynep.sahintas@fbe.karaelmas.edu.tr (Z.Ş.)

**Keywords:** antibacterial activity, DNA interaction, green synthesis, non-intercalative binding, phenolic-rich capping agents, *Salvia tomentosa*

## Abstract

This study reports the green synthesis of silver nanoparticles (AgNPs) using *Salvia tomentosa* L. leaf extract, and evaluates their physicochemical characteristics and biointerfacial performance, including DNA interaction, antibacterial activity, and antioxidant capacity. AgNP formation was confirmed by UV-Vis spectroscopy through a surface plasmon resonance band at 472 nm. SEM imaging showed predominantly spherical particles with sizes of 30–80 nm and a zeta potential of −17.3 mV, and EDX verified the elemental presence of silver. FTIR spectra indicated that plant-derived biomolecules, particularly phenolics, contributed to the reduction and capping/stabilization of AgNPs. XRD analysis confirmed a crystalline face-centered cubic structure. The AgNPs exhibited moderate, spontaneous binding to DNA (Kb ≈ 1.07 × 10^4^ M^−1^), characterized by pronounced hyperchromism without evidence of intercalation. Competitive fluorescence assays supported a predominantly non-intercalative, surface-associated interaction with minor groove perturbation, while agarose gel electrophoresis indicated preserved plasmid integrity and no extensive strand cleavage. Collectively, these results suggest reversible and structurally non-destructive AgNP–DNA complexation, indicating their potential for nucleic acid-related nano-biointerface studies, while further investigations are required to evaluate their suitability for biomedical applications. The biosynthesized AgNPs showed enhanced antibacterial activity against Gram-positive (*Bacillus cereus*) and Gram-negative (*Pantoea agglomerans*) bacteria compared with the leaf extract, whereas AgNO_3_ produced the strongest immediate effect, consistent with rapid Ag^+^ release. Antioxidant activity assessed by DPPH and ABTS assays showed strong radical-scavenging activity for the extract, in line with its high total phenolic content (206.2 mg GAE/g). Although AgNPs displayed lower phenolic content (164.2 mg GAE/g) and reduced antioxidant activity than the extract, they retained moderate scavenging capacity, indicating effective surface functionalization by phytochemicals. Overall, *S. tomentosa* leaf extract-capped AgNPs combine defined physicochemical features with non-destructive DNA association and antibacterial efficacy, underscoring their promise as phytochemical-functionalized nano-biointerfaces for antimicrobial and related biointerface applications.

## 1. Introduction

Nanotechnology enables the manipulation and assembly of materials at the nanometer scale, allowing the development of nanostructures with size-dependent physicochemical properties. In particular, metal and metal oxide nanoparticles exhibit distinctive optical and electronic behaviors as well as pronounced biological interactions, which underpin their applications across electronics, biotechnology, pharmacology, cosmetics, food-related technologies, diagnostics, biosensing, and materials science [1,2]. Accordingly, nanoscience has rapidly evolved into a major interdisciplinary field dedicated to the rational design and fabrication of functional nanomaterials. Nanoparticles are commonly produced via physical and chemical routes, including sol–gel processing, electrochemical deposition, lithography, mechanical milling, chemical vapor deposition, and thermal decomposition. However, conventional approaches often require toxic reagents, high energy input, and harsh reaction conditions, which can generate environmentally persistent by-products and raise concerns regarding biocompatibility—particularly when biomedical or biointerface applications are targeted [3]. These limitations have accelerated interest in green synthesis strategies that employ biological systems as sustainable and environmentally benign alternatives [4].

Various approaches have been developed for the synthesis of silver nanoparticles, including physical, chemical, and biological methods [5,6] [7,8]. Physical methods, such as evaporation–condensation and laser ablation, generally enable the production of highly pure nanoparticles with controlled morphology; however, they often require expensive instrumentation and high energy consumption. Chemical synthesis methods are widely preferred because of their simplicity, high yield, and ability to control particle size and shape, yet they frequently involve hazardous reducing agents and stabilizers that may generate toxic residues and limit biomedical applicability. In contrast, biological methods, particularly plant-mediated synthesis, provide an eco-friendly, cost-effective, and sustainable alternative due to the presence of natural reducing and stabilizing agents such as phenolics, flavonoids, and proteins [9]. Among these, plant-mediated synthesis is particularly attractive because it eliminates the need for toxic chemicals while enabling simultaneous reduction and stabilization of nanoparticles through naturally occurring phytochemicals. Nevertheless, biological synthesis may present certain limitations, including variability in phytochemical composition depending on plant source and extraction conditions, which can influence nanoparticle size, morphology, and stability [10]. Therefore, selecting plant species with well-characterized and phenolic-rich phytochemical profiles is crucial for obtaining reproducible and stable nanoparticles. Plant extracts contain diverse phytochemicals—such as phenolics, flavonoids, terpenoids, polysaccharides, alkaloids, and proteins—that can serve simultaneously as reducing agents and stabilizing/capping ligands during nanoparticle formation [11,12]. Importantly, the biomolecular layer formed on the nanoparticle surface plays a crucial role in determining nanoparticle stability, surface charge, colloidal properties, reactivity, and interactions with biological systems [13]. However, despite their ecological and biomedical advantages, plant-mediated synthesis approaches may suffer from variability in nanoparticle characteristics due to differences in phytochemical composition among plant species and extraction conditions [14]. By shaping the biological identity of nanoparticles rather than their original core structure, this structure can influence cellular internalization, biodistribution, toxicity, and overall biological activity [15]. Therefore, identifying plant sources with rich and relatively stable phytochemical profiles is essential for improving the reproducibility and functional performance of biosynthesized nanoparticles.

Silver nanoparticles (AgNPs) are among the most extensively investigated metallic nanomaterials owing to their high surface area-to-volume ratio and pronounced surface plasmon resonance. These attributes, together with their broad-spectrum antimicrobial potential, have motivated their incorporation into wound dressings, medical-device coatings, food-packaging materials, and drug-delivery platforms [16,17]. Mechanistically, AgNPs and released Ag^+^ ions may disrupt microbial membranes, induce oxidative stress, and interact with intracellular components, including nucleic acids, thereby contributing to growth inhibition and cell death [18].

In parallel, safe and efficient nucleic acid delivery remains a central challenge in gene therapy and gene engineering applications, since DNA is polyanionic and susceptible to enzymatic degradation in biological environments. Nanoparticle-based carriers offer key advantages, including tunable size, large specific surface area, and surface functionalization that can promote complexation with nucleic acids and improve stability [19,20]. AgNPs have therefore attracted interest as potential nucleic-acid delivery candidates, and previous reports indicate that AgNPs can associate with DNA primarily via electrostatic interactions and surface adsorption, in some cases providing partial protection against nuclease degradation [21,22]. Nevertheless, given the potential cytotoxicity associated with silver-based nanomaterials, detailed clarification of AgNP-DNA interaction modes and their structural consequences remains essential [23,24,25].

The genus Salvia (Lamiaceae) comprises approximately 1000 species worldwide and shows substantial diversity and endemism in the flora of Türkiye [26]. Several Salvia species are valued for medicinal and aromatic uses, with applications spanning traditional medicine, cosmetics, and food preservation. *Salvia tomentosa* L. has been used in Turkish folk medicine and has been associated with antibacterial, anti-inflammatory, and analgesic properties [27,28,29]. Phytochemical studies further indicate that *S. tomentosa* is rich in phenolic acids, flavonoids, diterpenoids, and essential oil components, which are considered important contributors to the reduction of silver ions and the stabilization of the resulting nanoparticles during green synthesis processes [26,27,28,29,30,31]. Such phenolic-rich profiles make *S. tomentosa* a promising source of natural reducing and capping agents for plant-mediated nanoparticle synthesis [32,33]. In addition, compared with other extensively investigated *Salvia* species, studies focusing on the nanoparticle synthesis potential of *S. tomentosa* remain limited. This lack of detailed investigation highlights the novelty of the present study and supports the selection of *S. tomentosa* as a promising natural source for the biosynthesis of functional phytochemical-capped AgNPs.

This study aims to investigate the feasibility of using a phenolic-rich *S. tomentosa* leaf extract as a reducing and capping agent for the green synthesis of AgNPs. The central hypothesis is that plant-derived phytochemical-capped AgNPs not only exhibit antimicrobial and antioxidant activities but can also engage in a controlled and reversible interaction with DNA, highlighting their potential relevance for delivery-related and other biointerface-oriented applications. Accordingly, the biosynthesized AgNPs were subjected to comprehensive physicochemical characterization, and their biointerface performance—including DNA binding behavior, antibacterial activity, and antioxidant capacity—was evaluated using an integrated approach.

## 2. Materials and Methods

### 2.1. Plant Material Collection and Aqueous Extract Preparation

Fresh leaves of *Salvia tomentosa* L. were collected in May from the experimental garden of the Department of Biology, Zonguldak Bülent Ecevit University (41.45124° N, 31.76037° E), Zonguldak, Turkey. The freshly harvested aerial parts were weighed (10 g) and cut into small pieces. The plant material was washed three times under running tap water and subsequently three times with distilled water to remove surface contaminants. The cleaned samples were transferred into a 250 mL Erlenmeyer flask, and 100 mL of sterile distilled water was added. The mixture was incubated in a water bath at 80 °C with shaking at 180 rpm for 1 h [34]. After extraction, the suspension was filtered through Whatman No. 1 filter paper, and the filtrate volume was adjusted to 100 mL with distilled water. The aqueous leaf extract was stored at 4 ± 1 °C in the dark until use.

### 2.2. Green Synthesis of Silver Nanoparticles (AgNPs)

An aqueous solution of 1.96 mg L^−1^ AgNO_3_ was prepared. For AgNP biosynthesis, 10 mL of *S. tomentosa* leaf extract was mixed with 100 mL of 1.96 mg L^−1^ AgNO_3_ solution and incubated at 90 °C for 6 h. The initially pale-colored reaction mixture gradually turned dark green after approximately 1 h, indicating the formation of AgNPs. To isolate the biosynthesized AgNPs, the reaction mixture was centrifuged at 5000 rpm for 5 min. The pellet was washed three times to remove unreacted silver ions and loosely bound constituents. After washing, the recovered AgNPs were transferred onto a clean glass slide and air-dried at room temperature for 24 h prior to further analyses.

### 2.3. Characterization of Biosynthesized AgNPs

#### 2.3.1. UV-Vis Spectroscopy

The formation of biosynthesized AgNPs was monitored by UV-Vis spectroscopy using a Tetra T80+ UV/VIS spectrophotometer (PG Instruments), Zonguldak, Turkey. After mixing the *S. tomentosa* leaf extract with AgNO_3_, nanoparticle formation was first qualitatively assessed by visual observation of the characteristic color change. Subsequently, absorption spectra were recorded in the range of 250–650 nm to determine the surface plasmon resonance (SPR) band of AgNPs. Measurements were performed using quartz cuvettes, and distilled water was used as the blank to zero the instrument [35].

#### 2.3.2. SEM Imaging and EDX Analysis

The morphology and size distribution of AgNPs were examined using a QUANTA 450 field-emission gun scanning electron microscope (FEG-SEM), Zonguldak, Turkey. Prior to imaging, the biosynthesized AgNPs were centrifuged at 5.000 rpm for 5 min. The resulting pellet was washed three times with distilled water to remove unreacted Ag (I) ions and residual plant constituents, then re-dispersed in distilled water by sonication for 5 min. The dispersion was passed through a 0.22 µm syringe filter. A 3 µL aliquot of the filtered sample was deposited onto a carbon tape on an aluminum stub and dried at room temperature for 15 min. Elemental composition was confirmed by energy-dispersive X-ray spectroscopy (EDX) performed on the same instrument.

#### 2.3.3. FTIR Spectroscopy

Fourier transform infrared (FTIR) spectroscopy was conducted using a PerkinElmer Spotlight 400 FTIR/FT-NIR spectrometer, Zonguldak, Turkey, to identify functional groups from the *S. tomentosa* extract involved in Ag^+^ reduction and nanoparticle capping. Spectra were collected in attenuated total reflectance (ATR) mode over the range of 400–4000 cm^−1^ with a resolution of 4 cm^−1^. For sample preparation, biosynthesized AgNPs were centrifuged at 5.000 rpm for 5 min and washed three times with distilled water to remove residual Ag (I) ions and extract components. The washed samples were then dried at 60 °C for 48 h prior to analysis [36].

#### 2.3.4. XRD Analysis

Phase formation, purity, and crystalline structure of the biosynthesized AgNPs were determined by X-ray diffraction (XRD) using a Panalytical Empyrean diffractometer (Zonguldak, Turkey) with Cu Kα radiation (λ = 1.54 Å). Diffractograms were recorded over a 2θ range of 20–80° at a scan speed of 0.1° min^−1^, step size of 0.02°, tube voltage of 45 kV, and tube current of 40 mA. For XRD measurements, AgNPs were collected by centrifugation at 5.000 rpm for 5 min, washed three times with distilled water, and dried at room temperature for 2 days.

All SEM, EDX, FTIR, and XRD analyses were performed at the Science and Technology Application and Research Center (ARTMER), Zonguldak Bülent Ecevit University, Zonguldak, Turkey.

#### 2.3.5. Zeta Potential Analysis

The zeta potential of the AgNPs suspension was determined using Zetasizer Nano ZSP (Ankara, Turkey) at ambient temperature (approximately 25 °C). Laser Doppler electrophoresis was used to measure the zeta potential. Before analysis, the AgNP suspensions were diluted with deionized water to obtain a concentration of 0.1 mg mL^−1^.

### 2.4. DNA İnteraction Assays

#### 2.4.1. UV-Vis Absorption Titration

For absorption titration experiments, AgNPs were first dispersed in a minimal volume of DMSO and then diluted with buffer (100 mM KCl, 10 mM Tris–HCl, pH 7.5) to obtain a final AgNP concentration of 1 mg mL^−1^. Spectroscopic titrations were performed in a stoppered 10 mm quartz cuvette by keeping the AgNP concentration constant while adding increasing aliquots of calf thymus DNA (CT-DNA; stock concentration 1.25 mM). After each addition, the mixtures were equilibrated for 10 min, and the absorption spectra were recorded.

#### 2.4.2. Ethidium Bromide (EB) Displacement Assay

Competitive binding of AgNPs toward DNA in the presence of EB was investigated by fluorescence spectroscopy. CT-DNA was incubated with EB at a DNA:EB molar ratio of 50:1 for 30 min at 27 °C. Emission spectra were recorded after excitation at 427 nm. Subsequently, increasing volumes of AgNP dispersion (1 mg mL^−1^) were added to the preformed DNA–EB complex, and emission spectra were collected after each addition to evaluate EB displacement based on changes in fluorescence intensity.

#### 2.4.3. DAPI Displacement Assay

Minor-groove competition was further assessed using 4′,6-diamidino-2-phenylindole (DAPI). CT-DNA was incubated with DAPI at a DNA:DAPI molar ratio of 1:1 in buffer (100 mM KCl, 10 mM Tris-HCl, pH 7.5) for 30 min at 27 °C to form the DNA-DAPI complex. Fluorescence emission spectra were acquired with excitation at 358 nm. Increasing aliquots of AgNP dispersion (1 mg mL^−1^) were then added to the DNA-DAPI complex. After each addition, samples were equilibrated for 5–10 min, and spectra were recorded under identical conditions. Variations in fluorescence intensity were analyzed to assess the extent of DAPI displacement.

#### 2.4.4. Agarose Gel Electrophoresis (DNA Retardation/Plasmid Integrity)

AgNP-DNA interactions were further examined by agarose gel electrophoresis using negatively supercoiled pBR322 plasmid DNA. Samples containing 20 ng pBR322 and increasing amounts of AgNPs were prepared at AgNP:DNA ratios of 0:1, 0.1:1, 0.5:1, 1:1, 2:1, 5:1, 10:1, 15:1, 20:1, and 30:1 (*w*/*w*, AgNP/DNA). Mixtures were incubated at 38 °C for 1 h, mixed with 2.5 µL of 0.25% bromophenol blue loading dye, and electrophoresed on 1% (*w*/*v*) agarose gels in Tris-borate-EDTA (TBE) buffer at 35 V for 4 h. Gels were stained with EB and visualized under UV illumination.

### 2.5. Antibacterial Activity

The antibacterial activity of the biosynthesized AgNPs was evaluated against one Gram-positive (*Bacillus cereus*), one Gram-negative (*Pantoea agglomerans*), one Gram-positive ATCC 23213 *Staphylococcus aureus*, and one Gram-negative ATCC 25922 *Escherichia coli* bacterium. The tested strains were field isolates obtained from diseased tomato fruits. Antibacterial activity of the biosynthesized AgNPs (0.06 µg mL^−1^, 0.12 µg mL^−1^, 0.24 µg mL^−1^, 0.48 µg mL^−1^) was compared with that of *S. tomentosa* leaf extract, commercially available AgNP standards (0.02 mg L^−1^ 40 nm and 0.02 mg L^−1^ 60 nm), and 1.96 mg L^−1^ AgNO_3_ using the agar well diffusion method. Antibacterial efficacy was assessed by measuring the diameter of the inhibition zones (zone of inhibition, ZOI).

Briefly, bacterial inocula were prepared by culturing each strain in 5 mL of tryptic soy broth (TSB) at 37 ± 2 °C for 24 h. The cultures were adjusted to a 0.5 McFarland standard, and 100 µL of the suspension was evenly spread onto Mueller–Hinton agar plates [36,37,38]. Wells of 8 mm diameter were punched aseptically in the agar at approximately 2.5 cm intervals. Each well was filled with 100 µL of the test sample at an applied concentration of 1 µg µL^−1^, and plates were incubated at 37 ± 2 °C for 48 h. After incubation, the ZOI was measured (mm). Sterile distilled water was used as the negative control, and AgNO_3_ was used as the reference standard. All assays were performed in triplicate, and results were reported as the mean of three independent measurements.

### 2.6. Evaluation of Antioxidant Activities

#### 2.6.1. DPPH Radical Scavenging Assay

The DPPH (2,2-diphenyl-1-picrylhydrazyl) free-radical scavenging activity of the samples was determined according to the method of Sánchez–Moreno with minor modifications [39]. Briefly, different volumes of the aqueous AgNP and *S.tomentosa* leaf extract preparation (0.06 µg mL^−1^, 0.12 µg mL^−1^, 0.24 µg mL^−1^, 0.48 µg mL^−1^) were transferred into test tubes, and 2.0 mL of freshly prepared ethanolic DPPH solution was added to each tube. The mixtures were vortexed and incubated in the dark for 30 min at room temperature. The decrease in absorbance was then measured at 517 nm using a UV-Vis spectrophotometer (Tetra T80+ UV/VIS, PG Instruments) with quartz cuvettes. Butylated hydroxyanisole (BHA) was used as the reference antioxidant standard.

DPPH radical scavenging activity was calculated as shown in Equation (1):

Inhibition (%) = [(A_control − A_sample)/A_control] × 100(1)

where A_control is the absorbance of the DPPH solution without a sample, and A_sample is the absorbance in the presence of the tested sample.

#### 2.6.2. ABTS Radical Cation Decolorization Assay

The ABTS [2,2′-azinobis-(3-ethylbenzothiazoline-6-sulfonic acid)] radical cation scavenging activity was evaluated. The ABTS•^+^ radical cation was generated by mixing 6.5 mM ABTS solution with 2.45 mM potassium persulfate at a 1:1 (*v*/*v*) ratio and incubating the mixture in the dark at room temperature for 16 h until a dark green color developed. The ABTS•^+^ solution was then diluted with distilled water to obtain an absorbance of 0.6–0.8 at 734 nm [40].

For the assay, different volumes of the aqueous AgNP and *S.tomentosa* leaf extract preparation (0.06 µg mL^−1^, 0.12 µg mL^−1^, 0.24 µg mL^−1^, 0.48 µg mL^−1^) were added into test tubes, followed by 2.0 mL of freshly prepared aqueous ABTS•^+^ solution. The mixtures were vortexed and incubated in the dark for approximately 30 min at room temperature. Absorbance was measured at 734 nm using a UV-Vis spectrophotometer (Tetra T80+ UV/VIS, PG Instruments) with quartz cuvettes. BHA was used as the reference standard.

ABTS radical scavenging activity was calculated as shown in Equation (2):

Inhibition (%) = [(A_control − A_sample)/A_control] × 100(2)

### 2.7. Determination of Total Phenolic Content (TPC)

Total phenolic content (TPC) was estimated using the Folin–Ciocalteu colorimetric method based on the oxidation-reduction reaction of phenolic compounds, with minor modifications of the procedure described by Singleton et al. (1999) [41]. Briefly, 100 µL of each sample (AgNPs and extract) was mixed with 100 µL of Folin–Ciocalteu reagent (Merck) and allowed to react for 5 min at room temperature. Subsequently, 300 µL of 20% (*w*/*v*) Na_2_CO_3_ solution and 1580 µL of distilled water were added. The reaction mixture was incubated at room temperature for 30 min, and absorbance was measured at 765 nm using a UV-Vis spectrophotometer (Tetra T80+ UV/VIS, PG Instruments) with distilled water as the blank.

A gallic acid calibration curve was prepared using standard solutions at 15, 30, 60, 125, 250, and 500 mg L^−1^. TPC values were calculated from the calibration curve and expressed as mg gallic acid equivalents per g of extract (mg GAE/g extract).

TPC was calculated as shown in Equation (3):

TPC (mg GAE/g extract) = (A_sample − A_blank)/slope × DF(3)

where A_sample is the absorbance of the sample reaction mixture, A_blank is the absorbance measured for distilled water, slope is obtained from the gallic acid calibration curve, and DF is the dilution factor.

### 2.8. Statistical Analysis

Antibacterial assays were conducted in three independent replicates, and inhibition-zone diameters were reported as the mean of three measurements. Spectrophotometric assays (DPPH, ABTS, and TPC) were measured in triplicate. All experiments were performed in triplicate, and results are presented as mean ± SD.

## 3. Results and Discussion

### 3.1. Characterization of Silver Nanoparticles

**Figure 1:**
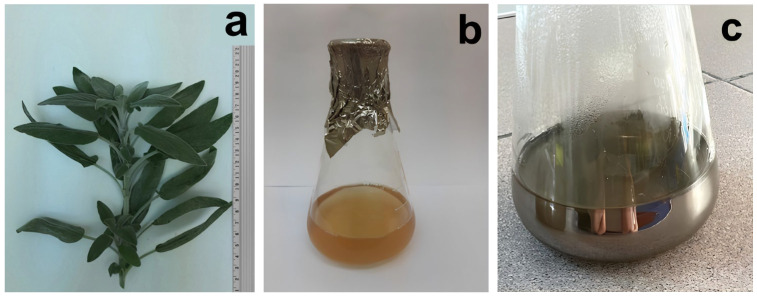
Silver nanoparticle formation during the green synthesis using *Salvia tomentosa* L. leaf extract. (**a**) *Salvia tomentosa* L. leaf, (**b**) reaction mixture, (**c**) the color change in the reaction mixture, dark green with a mirror-like appearance after 6 h of incubation.

Approximately 1 h after the aqueous *S. tomentosa* L. leaf extract was added to the silver nitrate solution, the reaction mixture changed in color from clear to dark green and exhibited a mirror-like appearance (Figure 1). This visible transformation indicates the reduction in Ag^+^ ions and the formation of silver nanoparticles (AgNPs) in the colloidal suspension. Similar color changes associated with plant-mediated AgNP formation have been reported in previous studies, supporting our observations [35]. To further confirm AgNP synthesis and elucidate their physicochemical features, the biosynthesized nanoparticles were characterized using UV-Vis spectroscopy, SEM, EDX, FTIR, and XRD analyses.

**Figure 2:**
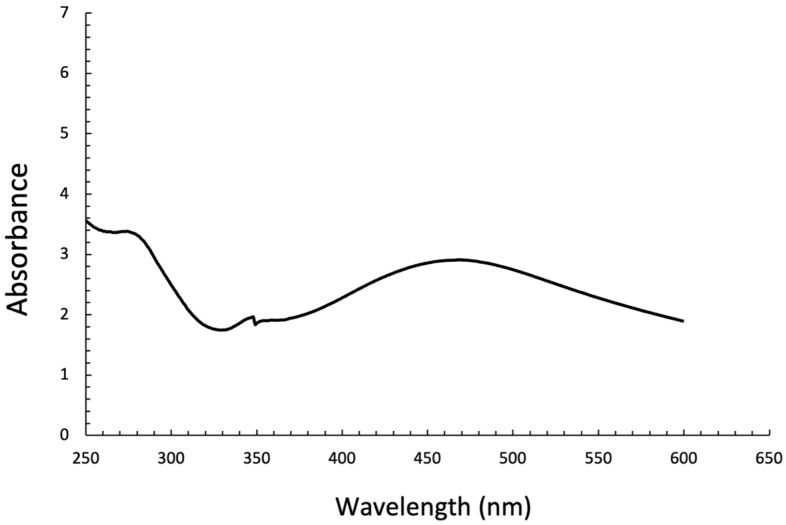
UV-Vis absorption spectrum of silver nanoparticles biosynthesized using *Salvia tomentosa* L. extract, showing a characteristic surface plasmon resonance (SPR) band centered at approximately 472 nm.

The UV-Vis absorption spectrum of the AgNPs synthesized using *S. tomentosa* L. leaf extract recorded after 6 h in the range of 250–650 nm is shown in Figure 2. The AgNP dispersion exhibited a distinct maximum absorbance at 472 nm. The presence of this band is characteristic of AgNP formation and is attributed to the surface plasmon resonance (SPR) of conduction electrons at the nanoparticle surface [35]. The single, well-defined SPR band suggests the formation of predominantly spherical AgNPs with a relatively narrow size distribution.

The UV-Vis spectrum of silver nanoparticles biosynthesized using *Salvia tomentosa* L. extract exhibited a characteristic surface plasmon resonance (SPR) band centered at approximately 472 nm. The SPR peak appeared as a single but relatively broad band, which is indicative of spherical nanoparticles with a heterogeneous size distribution [3,4,5,6,9,10,11,12,13,14,15,16,17,18,19,20,21,22,23,24,25,26,27,28,29,30,31,32,33,34,35]. This observation is in good agreement with SEM analysis, which revealed predominantly spherical AgNPs with particle sizes ranging from 30 to 80 nm.

It is documented that an increase in nanoparticle size leads to a red shift and broadening of the SPR band due to increased polydispersity and size dispersion effects [42]. Accordingly, the red-shifted and broadened SPR band observed in this study can be attributed to the relatively large particle size and polydisperse nature of the biosynthesized AgNPs. Additionally, the absorption observed in the lower wavelength region (250–350 nm) may be associated with phytochemical constituents of the *S. tomentosa* extract adsorbed on the nanoparticle surface, acting as capping agents and contributing to nanoparticle stabilization [43]. Overall, the UV-Vis spectral features are consistent with successful green synthesis of AgNPs and are well supported by SEM findings. The broad SPR band reflects the polydisperse size distribution observed in SEM images rather than nanoparticle aggregation.

**Figure 3:**
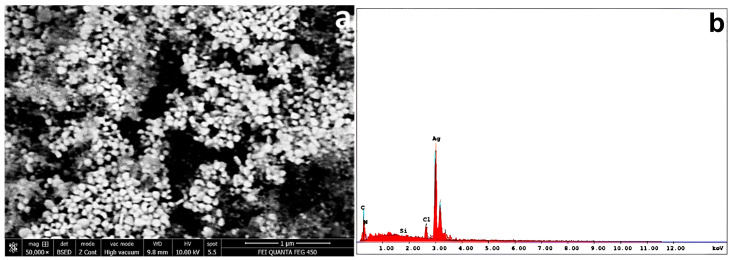
(**a**) SEM image of silver nanoparticles biosynthesized using *Salvia tomentosa* L. extract, revealing spherical morphology and particle sizes of approximately 30–80 nm. (**b**) Corresponding EDX spectrum confirming the elemental composition of the synthesized silver nanoparticles.

SEM micrographs showed that the biosynthesized AgNPs were predominantly spherical (Figure 3a). The corresponding size distribution indicated particle diameters mainly in the range of approximately 30–80 nm. The elemental composition of the AgNPs was further examined by energy-dispersive X-ray (EDX) analysis. The EDX spectrum displayed a strong and distinct signal at ~3 keV, which is characteristic of metallic silver and confirms successful AgNP formation. In addition, minor signals corresponding to C, O, N, Cl, and Si were detected (Figure 3b). The C, O, and N peaks are plausibly associated with plant-derived organic constituents adsorbed on the nanoparticle surface, supporting phytochemical capping/stabilization. The trace Cl and Si signals may originate from residual inorganic components and/or the supporting substrate/sample holder used during SEM-EDX measurements. The dominance of the Ag signal and the comparatively weak intensities of the other elements indicate that the nanoparticles are primarily composed of silver, with surface functionalization by biomolecules. Overall, the EDX results support the successful biosynthesis of AgNPs and are consistent with previous reports on plant-mediated AgNP synthesis [3].

**Figure 4:**
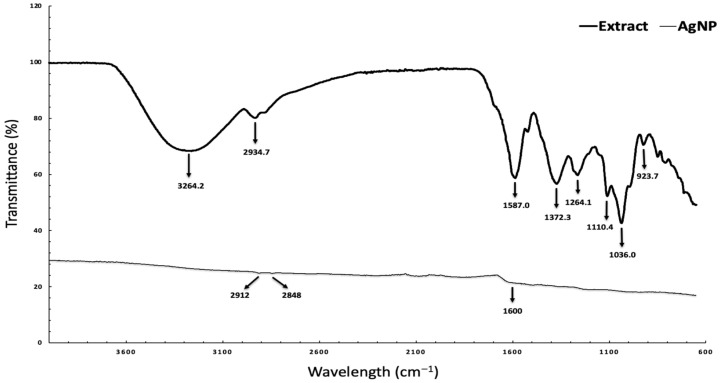
FTIR spectrum of *Salvia tomentosa* L. extract and silver nanoparticles biosynthesized using *S. tomentosa* L. extract, indicating the presence of functional groups associated with plant-derived biomolecules involved in the reduction and stabilization of silver nanoparticles.

FTIR spectroscopy was employed to identify the functional groups involved in the biosynthesis and stabilization of silver nanoparticles mediated by *S. tomentosa* L. extract. The comparison between the FTIR spectra of the plant extract and the synthesized AgNPs reveals shifts in characteristic peaks, indicating the involvement of phytochemicals in the reduction, capping, and stabilization processes. The broad absorption band observed in the range of 3264.2 and 2934.7 cm^−1^ corresponds to O–H and N–H stretching vibrations, indicating the presence of phenolic compounds, flavonoids, and proteins in *S. tomentosa* L. extract, while the band at approximately 1587, 1372.3, 1264.1, 1110.4, 1036, and 923.7 cm^−1^ can be assigned to C=O or aromatic C=C stretching vibrations (Figure 4). These biomolecules are known to play a crucial role in the reduction in Ag^+^ ions and subsequent stabilization of the formed nanoparticles [44].

The bands detected around 2912–2848 cm^−1^ are attributed to C–H stretching vibrations of aliphatic groups, while the absorption near 1600 cm^−1^ can be assigned to C=O or aromatic C=C stretching, suggesting the involvement of proteinaceous and phenolic components. Additional bands in the region of 1000–1100 cm^−1^ correspond to C–O stretching vibrations, further supporting the presence of plant-derived organic compounds on the nanoparticle surface (Figure 4). The FTIR results collectively confirm that phytochemicals present in *S. tomentosa* extract function as reducing and capping agents, contributing to the successful green synthesis and stabilization of silver nanoparticles. The FTIR findings are consistent with EDX results, which revealed the presence of carbon and oxygen, further confirming the surface functionalization of AgNPs by plant-derived biomolecules. These observations are consistent with previously reported studies on green-synthesized AgNPs, where shifts in FTIR bands were attributed to the involvement of phenolic and flavonoid compounds in both reduction and surface stabilization.

The comparison of FTIR spectra between *S. tomentosa* extract and the synthesized AgNPs reveals noticeable shifts and changes in peak intensities, indicating the involvement of phytochemicals in nanoparticle formation. For instance, the broad O–H/N–H stretching band observed in the extract is slightly shifted and reduced in intensity in the AgNP spectrum, suggesting the participation of phenolic and proteinaceous compounds in the reduction and stabilization processes. Similarly, changes in the band around 1600 cm^−1^, associated with C=O and aromatic C=C stretching, further support the interaction of these functional groups with the nanoparticle surface. These observations are consistent with previously reported studies on green-synthesized AgNPs, where shifts in FTIR bands were attributed to the involvement of phenolic and flavonoid compounds in both reduction and surface stabilization [40,41,42,43,44,45].

**Figure 5:**
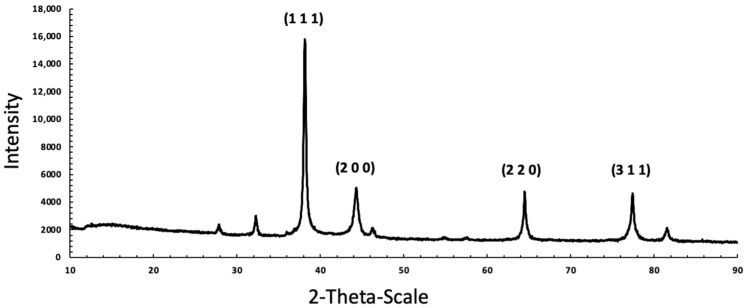
XRD pattern of silver nanoparticles biosynthesized using *Salvia tomentosa* L. extract, showing characteristic diffraction peaks corresponding to the face-centered cubic (fcc) crystalline structure of metallic silver.

The crystalline nature of the biosynthesized AgNPs was investigated using X-ray diffraction (XRD) analysis. The XRD pattern exhibited distinct diffraction peaks at 2θ values of approximately 38°, 44°, 64°, and 77°, which correspond to the (111), (200), (220), and (311) crystallographic planes, respectively (Figure 5). These peaks are characteristic of face-centered cubic (fcc) crystalline silver and are in good agreement with standard JCPDS data (No. 04-0783).

The prominent intensity of the (111) reflection indicates a preferential orientation along this plane, which is commonly observed in biosynthesized AgNPs and suggests enhanced structural stability. The absence of additional diffraction peaks related to silver oxide or other impurity phases confirms the high crystalline purity of the synthesized nanoparticles. Overall, the XRD results confirm the successful formation of crystalline metallic silver nanoparticles through a green synthesis route mediated by *S. tomentosa* L. extract.

The Scherer–Debye equation was used to calculate the average crystallite size of AgNPs as shown in Equation (4):

D = (K × λ)/β × cosθ(4)

D = the size of the crystal, its unit is equal to λ unit, usually angstrom or nm;

λ = the X-ray wavelength, used the K-Alpha1 wavelength;

K = a dimensionless shape factor, with a value close to unity;

Β = the full width at half maximum;

θ = the peak position on the horizontal axis of the diffraction pattern, which, if the horizontal axis is 2θ. It should be divided into two to get θ.

The average crystallite size of the biosynthesized silver nanoparticles was calculated using the Scherrer equation based on the (111), (200), (220), and (311) diffraction peaks. The crystallite sizes were estimated to be approximately 33, 34, 73, and 57 nm for the respective planes, resulting in an average crystallite size of about 49 nm. The variation in crystallite size among different crystallographic planes may be attributed to anisotropic crystal growth and preferential orientation during the green synthesis process.

The average crystallite size obtained from XRD analysis is smaller than the particle size range observed in SEM images (30–80 nm), indicating that individual nanoparticles may consist of multiple crystalline domains. Such differences between crystallite size and particle size are commonly reported for plant-mediated synthesis of silver nanoparticles and can be explained by particle aggregation and surface capping by phytochemicals present in *S. tomentosa* L. extract. The use of multiple diffraction peaks provides a more reliable estimation of crystallite size and further confirms the polycrystalline nature of the synthesized AgNPs.

The choice of plant extract plays a crucial role in determining the physicochemical properties and biological activities of the synthesized nanoparticles. Different plant species contain distinct profiles of bioactive compounds, which influence nanoparticle size, morphology, stability, and functional performance. For instance, extracts rich in phenolic compounds tend to produce smaller and more stable nanoparticles, while variations in flavonoid and protein content may affect capping efficiency and bioactivity. Therefore, investigating different plant sources is essential for optimizing nanoparticle characteristics and expanding their potential applications.

Zeta potential analysis revealed an average surface charge of −17.3 mV for the biosynthesized AgNPs, indicating moderate colloidal stability of the nanoparticle suspension. The negative surface charge suggests the adsorption of negatively charged phytochemicals derived from the *S. tomentosa* extract onto the nanoparticle surface, contributing to electrostatic stabilization [35,36,37]. The combined UV-Vis, SEM, EDX, FT-IR, XRD, and zeta potential analyses collectively confirm the successful green synthesis, crystalline structure, surface functionalization, and morphological characteristics of *S. tomentosa*-mediated silver nanoparticles.

### 3.2. Investigation of the Interaction of AgNP with DNA

#### 3.2.1. UV-Vis Titration

UV-Vis absorption spectroscopy is widely employed to elucidate the binding interactions between small molecules and DNA. Different binding modes, including electrostatic attraction, groove binding, and intercalation between DNA base pairs, give rise to distinct spectral changes. In particular, intercalative binding is typically characterized by a decrease in absorbance (hypochromism) accompanied by a bathochromic shift in the maximum absorption wavelength [46]. In contrast, when small molecules associate with DNA through groove binding or electrostatic interactions with the nucleobases or phosphate backbone, an increase in absorbance (hyperchromism) is generally observed [47].

**Figure 6:**
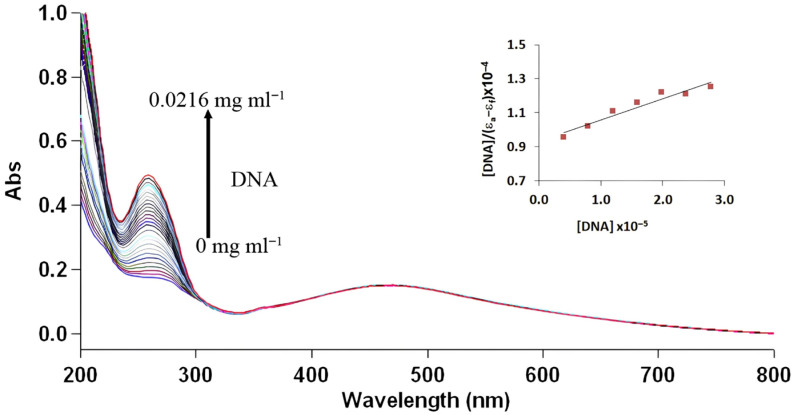
UV-vis spectra of silver nanoparticles (1 mg mL^−1^ in a buffer 100 mM KCl, 10 mM Tris, pH 7.5) in the presence of different concentrations of ct-DNA (2 µL of 1.25 mM stock solution).

In order to clarify the interaction mechanism between the AgNP and ctDNA in aqueous media, the electronic absorption spectra of AgNP were recorded in the presence of increasing concentrations of ctDNA (Figure 6).

The interaction between silver nanoparticles (AgNPs) and calf thymus DNA (ct-DNA) was characterized using UV–visible absorption titration, revealing an intrinsic binding constant (*K*_b_)—which can be calculated from the following Equation (5)—of approximately 1.07 × 10^4^ mg^−1^ mL.

[DNA]/(*ε*_A_ − *ε*_f_) = [DNA]/(*ε*_B_ − *ε*_f_) + 1/*K*_b_(*ε*_B_ − *ε*_f_) (5)

This value indicates a moderate affinity that aligns more closely with electrostatic or groove binding mechanisms rather than the high-affinity intercalation (10^5^–10^6^) typically observed with planar aromatic molecules. The magnitude of this constant suggests that while the AgNPs associate significantly with the DNA scaffold, they do not penetrate the hydrophobic interior of the double helix as deeply as classical intercalators do [48]. The thermodynamic spontaneity of the interaction was supported by the negative Gibbs free energy change (ΔG° ≈ −23 kJ/mol), confirming that the binding process occurs spontaneously under physiological conditions. However, the magnitude of ΔG° further supports a non-intercalative mechanism, as stronger π–π stacking interactions typically produce more negative free energy values.

A defining feature of this interaction is the massive hyperchromic effect, characterized by a roughly 220% increase in absorbance at approximately 270 nm. In the context of DNA biophysics, such pronounced hyperchromism signals a major disruption of the π-π* transitions within the nucleobases, usually resulting from the destabilization of base stacking and increased exposure of the bases to the surrounding solvent. Unlike classical intercalation, which is marked by hypochromism and bathochromic shifts due to enhanced π-π* overlap, the observed spectral profile strongly suggests that AgNPs induce a conformational unwinding or structural perturbation of the DNA helix [49].

Mechanistically, this association is likely driven by surface-mediated interactions, including electrostatic attraction between the nanoparticle surface—or its associated cations—and the negatively charged phosphate backbone of the DNA. This process, potentially complemented by groove binding facilitated by surface ligands, leads to a significant distortion of the double helix. The unusually high hyperchromic response further implies that the structural impact may involve localized disruption of hydrogen bonding or the influence of released Ag^+^ ions interacting directly with the bases, both of which would contribute to the overall destabilization of the macromolecule [50].

#### 3.2.2. Competitive Emission Titration with EB and DAPI

**Figure 7:**
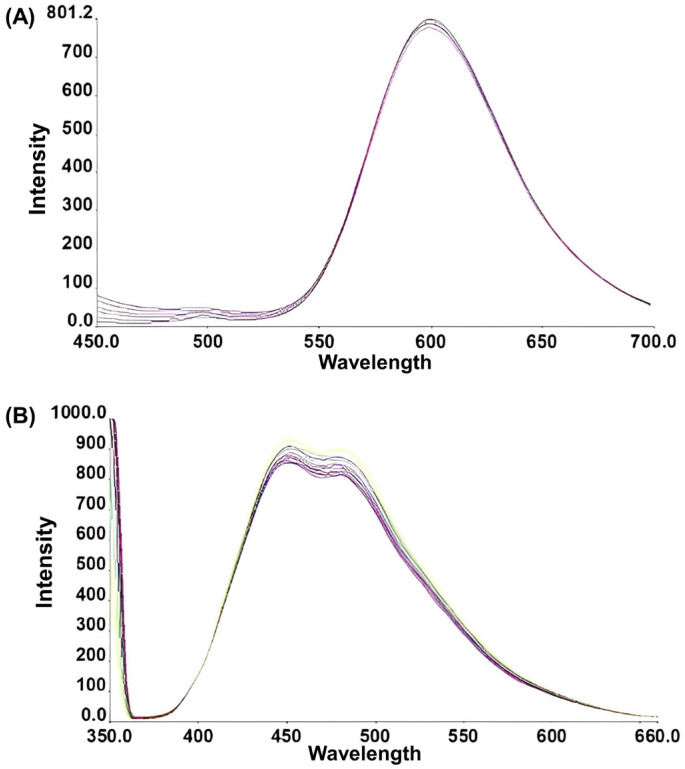
The change in the emission intensity of the EB-DNA mixture (**A**) and DAPI-DNA mixture (**B**) with the addition of silver nanoparticles in fixed portions (5 µL of 1 mg mL^−1^ solution).

To further clarify the binding mode of AgNPs toward DNA, competitive fluorescence displacement assays were conducted using ethidium bromide (EB) (Figure 7A) and DAPI (Figure 7B) as site-selective probes. The EB–DNA system exhibited no significant change in fluorescence intensity upon incremental addition of AgNPs. Since EB strongly fluoresces only when intercalated between DNA base pairs, the absence of fluorescence quenching indicates that AgNPs do not effectively displace EB from its intercalation sites. This observation strongly suggests that AgNPs lack a classical intercalative binding mode.

In contrast, titration of AgNPs into the DAPI-DNA complex resulted in an approximately 10% decrease in fluorescence intensity. Given that DAPI binds preferentially within the minor groove of AT-rich regions, partial quenching of DAPI fluorescence implies that AgNPs may interact within or near the groove environment. However, the relatively modest decrease (~10%) indicates that this interaction is not strongly competitive and does not fully displace DAPI from its binding site.

Taken together, the absence of EB displacement combined with mild DAPI fluorescence reduction supports a non-intercalative binding mechanism, most consistent with weak groove association and/or electrostatic interaction with the phosphate backbone. The limited degree of DAPI quenching further suggests that AgNP binding does not involve specific high-affinity groove occupation, but rather induces subtle conformational perturbations that partially affect groove accessibility.

These findings are consistent with the moderate binding constant (Kb ≈ 10^4^ mg^−1^ mL) obtained from UV-Vis titration and the pronounced hyperchromism observed in absorption spectra. While the hyperchromic effect indicates structural perturbation of DNA base stacking, the competitive fluorescence results demonstrate that such perturbation does not arise from base pair intercalation. Instead, the data collectively suggest that AgNPs interact at the DNA surface, likely through electrostatic attraction or shallow groove association, leading to partial destabilization of the double helix without deep insertion between base pairs.

Overall, the competitive emission data corroborate a surface-associated, non-intercalative binding mode of AgNPs with DNA, characterized by moderate affinity and limited but measurable perturbation of the minor groove region.

#### 3.2.3. DNA Retardation Study by Agarose Gel Electrophoresis

To investigate whether AgNPs induce conformational alterations or complex formation with plasmid DNA, a DNA retardation assay was performed using pBR322. Samples containing 20 ng of pBR322 DNA were incubated with increasing concentrations of AgNPs (AgNP/DNA ratios of 0:1, 0.1:1, 0.5:1, 1:1, 2:1, 5:1, 10:1, 15:1, 20:1, and 30:1 mg mL^−1^) at 38 °C for 1 h prior to electrophoresis.

**Figure 8:**
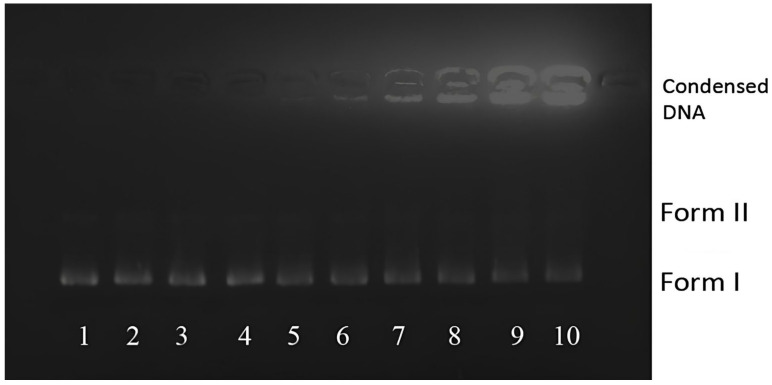
The electrophoresis results of the different mixtures containing 10 µL of pBR322 plasmid DNA (0.02 mg mL^−1^) and 10 µL of AgNP with different concentrations (lanes 1–10 (r = w_copolymer_/w_DNA_); 0:1, 0.1:1, 0.5:1, 1:1, 2:1, 5:1, 10:1, 15:1, 20:1, and 30:1 mg mL^−1^ respectively) at 38 °C for 1 h.

The gel electrophoresis profiles (Figure 8) demonstrated that DNA migration was largely preserved across all tested nanoparticle ratios. No complete retardation or immobilization of DNA within the wells was observed, even at the highest AgNP concentration. This indicates that AgNPs do not induce strong condensation or formation of highly stable nanoparticle–DNA aggregates capable of preventing electrophoretic mobility.

Importantly, no significant smearing or fragmentation bands were detected, suggesting that under the applied incubation conditions (38 °C, 1 h), AgNP exposure did not result in extensive strand breaks or degradation of the phosphodiester backbone. The preservation of discrete plasmid bands implies that the structural integrity of pBR322 DNA remains largely intact.

At higher AgNP/DNA ratios, a slight reduction in band intensity and minor accumulation near the loading wells were observed. This behavior may reflect partial surface complexation or local charge neutralization effects, likely arising from electrostatic interactions between AgNP surfaces and the negatively charged DNA backbone. Such interactions can moderately alter electrophoretic mobility without causing complete DNA condensation or irreversible binding.

These electrophoretic findings are consistent with the spectroscopic results obtained from UV-Vis and competitive fluorescence assays. The absence of EB displacement excludes classical intercalation, while the modest DAPI fluorescence reduction (~10%) suggests limited groove association. Together with the moderate binding constant (Kb ≈ 10^4^ mg^−1^ mL), the gel data support a non-intercalative, surface-associated interaction mechanism characterized by reversible electrostatic binding rather than strong structural immobilization or cleavage.

The observed non-intercalative and largely surface-associated AgNP–DNA interaction can be rationalized by considering the phytochemical formed during plant-mediated synthesis. FTIR signatures (broad O–H/N–H and C–O-related bands) together with EDX-detected light elements (C, O, and N) indicate that the AgNP surface is coated with phenolic- and protein/polysaccharide-like biomolecules originating from the *S. tomentosa* extract. Such a “phytochemical capping layer” is expected to govern the biointerface by (i) modulating surface charge and polarity, (ii) providing hydrogen-bond donors/acceptors, and (iii) presenting aromatic and carbonyl functionalities capable of weak π–π and dipole interactions with nucleobases. In aqueous media, DNA complexation is therefore likely driven by a combination of electrostatic attraction between the DNA phosphate backbone and positively polarized/ion-bridged sites on the capped AgNP surface, together with multivalent hydrogen bonding and van der Waals contacts at the hydration layer. This interfacial model is consistent with the pronounced hyperchromism observed in UV–Vis titrations and with competitive fluorescence assays, which indicated limited perturbation of base stacking and the groove environment rather than classical intercalation. Moreover, the preservation of plasmid topology in agarose gel electrophoresis supports a reversible, structurally non-destructive association, suggesting that the phytochemical may attenuate direct metal–DNA contact and mitigate strand cleavage under the tested conditions. These findings highlight that plant-derived surface functionalization is not merely a stabilizing feature but a key determinant of AgNP–DNA biointerface behavior.

Overall, the DNA retardation assay confirms that although AgNPs interact with plasmid DNA and may induce conformational modulation, they do not cause substantial strand breakage or strong nanoparticle-mediated condensation under the experimental conditions employed.

### 3.3. Antibacterial Activity by the Agar Well Diffusion Method

**Figure 9:**
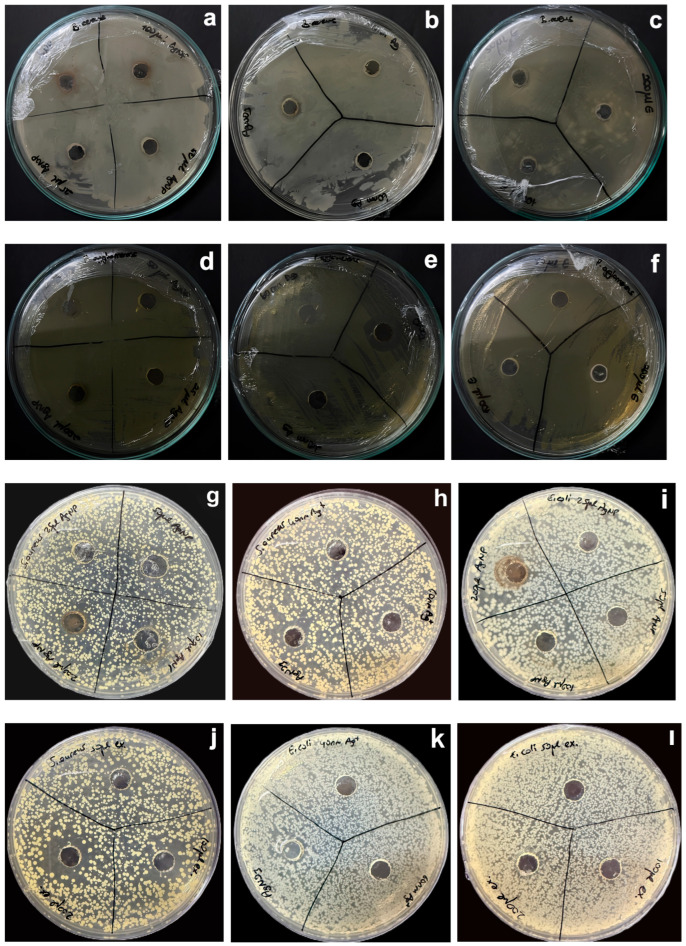
Agar well diffusion plates showing the antibacterial activity of *S. tomentosa* L. leaf extract, biosynthesized AgNPs, AgNO_3_, and commercial AgNP standards (40 nm and 60 nm) against: *Bacillus cereus* (Gram-positive) (**a**–**c**), *Pantoea agglomerans* (Gram-negative) (**d**–**f**), *Staphylococcus aureus* ATCC 23213 (Gram-positive) (**g**–**i**), ATCC 25922 *Escherichia coli* (Gram-negative) (**j**–**l**).

The antimicrobial activity of the biosynthesized AgNPs, AgNO_3_, commercial Ag^+^ ions (40 and 60 nm), and *S. tomentosa* L. leaf extract against pathogenic microorganisms is presented in Figure 9. Bacterial growth was observed against *B. cereus* for AgNPs (0.7 mm), AgNO_3_ (0.7 mm), 40 nm Ag^+^ (0 mm), 60 nm Ag^+^ (0 mm), and the plant extract (0 mm). Similarly, against *P. agglomerans*, inhibition was observed for AgNPs (0.3 mm) and AgNO_3_ (1.7 mm), whereas no antibacterial effect was detected for 40 nm Ag^+^, 60 nm Ag^+^, and the extract. For *S. aureus* ATCC 23213, inhibition zones were recorded for AgNPs (0.2 mm), AgNO_3_ (0.7 mm), and the plant extract (1.2 mm), whereas the commercial AgNPs showed no detectable activity. In the case of *E. coli* ATCC 25922, weak inhibition was observed for AgNPs (0.2 mm) and AgNO_3_ (0.3 mm), while no antibacterial effect was detected for the commercial AgNPs or the extract. The relatively lower antibacterial activity of AgNPs compared to AgNO_3_ may be associated with the gradual release of Ag^+^ ions from the nanoparticle surface, which provides a more controlled but less immediate antimicrobial effect. The results clearly indicate that the *S. tomentosa* L. leaf extract alone did not exhibit sufficient antimicrobial activity against the tested microorganisms, suggesting that the bioactive compounds present in the extract were not effective at the applied concentration.

In contrast, the biosynthesized AgNPs demonstrated higher antibacterial activity against both Gram-positive (*Bacillus cereus*) and Gram-negative (*Pantoea agglomerans*) bacteria compared to the plant extract. This enhanced activity can be attributed to the nanoscale size, spherical morphology, and high surface area of AgNPs, which facilitate close interaction with bacterial cell membranes [51]. Among all tested samples, AgNO_3_ exhibited the highest antibacterial activity, which may be explained by the immediate release of Ag^+^ ions, known for their strong bactericidal properties.

The antibacterial activity of silver nanoparticles has been widely attributed to their ability to release Ag^+^ ions, generate reactive oxygen species (ROS), and disrupt bacterial cell membrane integrity. Previous studies have reported that green-synthesized AgNPs interact with thiol-containing proteins in bacterial membranes, leading to increased membrane permeability and subsequent cell death [22,23,24,25,26,27,28,29,30,31,32,33,34,35,36,37,38,39,40,41,42,43,44,45,46,47,48,49,50,51,52].

Furthermore, particle size and morphology play a critical role in determining antimicrobial efficacy, as smaller and spherical nanoparticles provide a larger surface area for interaction with microbial cells [53]. In the present study, the spherical morphology and nanoscale dimensions (30–80 nm) of *S. tomentosa*–mediated AgNPs likely contributed to their antibacterial performance against both Gram-positive and Gram-negative bacteria.

Additionally, the difference in antibacterial susceptibility between Gram-positive and Gram-negative bacteria may be attributed to variations in cell wall structure. Gram-negative bacteria possess an outer membrane that can influence nanoparticle penetration, whereas Gram-positive bacteria have a thicker peptidoglycan layer, which may interact differently with silver nanoparticles. These findings are consistent with previous reports indicating that green-synthesized AgNPs exhibit moderate to strong antibacterial activity depending on particle size, surface chemistry, and microbial strain [54].

The higher antibacterial activity observed for AgNO_3_ compared to biosynthesized AgNPs can be attributed to the immediate availability and rapid diffusion of free Ag^+^ ions in the growth medium. AgNO_3_ readily dissociates in aqueous environments, allowing Ag^+^ ions to directly interact with bacterial cell walls, membrane proteins, and intracellular components, leading to rapid cellular damage and bacterial death [22].

In contrast, AgNPs act as a reservoir of silver ions, releasing Ag^+^ gradually through oxidative dissolution processes occurring at the nanoparticle surface [52]. This process involves the oxidation of metallic silver (Ag^0^) to Ag^+^ in the presence of dissolved oxygen and protons, typically represented as

Ag^0^ → Ag^+^ + e^−^

O_2_ + 4H^+^ + 4e^−^ → 2H_2_O

As a result, Ag^+^ ions are continuously generated and released into the surrounding medium. The kinetics of this process are governed by several environmental and physicochemical factors [55]. For instance, acidic pH conditions enhance silver oxidation and ion release, whereas higher ionic strength may promote aggregation and reduce the effective reactive surface area. Additionally, smaller nanoparticles exhibit faster dissolution rates due to their higher surface-to-volume ratio. Surface-bound phytochemicals, as identified by FTIR analysis, can further modulate this process by stabilizing the nanoparticle surface and slowing Ag^+^ release.

Therefore, AgNPs function as a sustained source of silver ions, resulting in a controlled but prolonged antibacterial effect compared to the rapid action of AgNO_3_. This controlled release behavior may contribute to reduced cytotoxicity and improved long-term antimicrobial performance.

### 3.4. Evaluation of Antioxidant Capacity Using DPPH and ABTS

**Figure 10:**
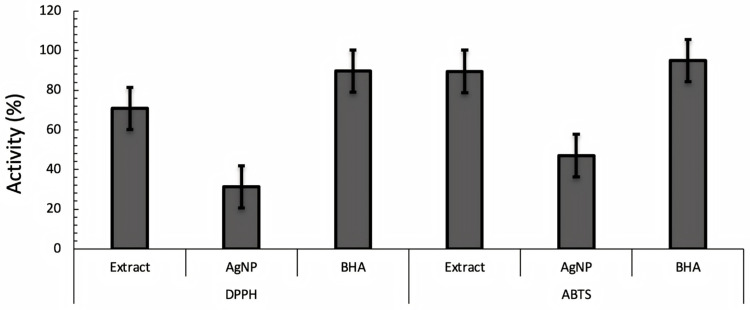
DPPH and ABTS radical scavenging activities of *S. tomentosa* leaf extract, biosynthesized silver nanoparticles, and BHA at 1 mg mL^−1^ concentration. Results are expressed as mean ± SD (*n* = 3).

The DPPH radical scavenging activity of *S. tomentosa* leaf extract, biosynthesized AgNPs, and the standard antioxidant BHA at different concentrations is presented in Figure 10. The results demonstrated a clear concentration-dependent antioxidant activity for all tested samples. Among them, the *S. tomentosa* leaf extract exhibited significantly higher DPPH radical scavenging activity than the biosynthesized AgNPs at all tested concentrations. At 1 mg mL^−1^, the leaf extract showed a scavenging activity of 70.83%, whereas AgNPs exhibited a markedly lower activity of 31.25%.

The strong antioxidant activity of the leaf extract can be attributed to its rich content of phenolic compounds and flavonoids, which are well known for their hydrogen-donating ability and radical scavenging potential. These phytochemicals are capable of directly neutralizing DPPH radicals, resulting in higher inhibition percentages compared to AgNPs. Although the antioxidant activity of AgNPs was lower than that of the crude extract, the observed scavenging activity indicates that plant-derived biomolecules remain attached to the nanoparticle surface, as supported by FTIR analysis.

Compared to the synthetic antioxidant BHA, which exhibited the highest radical scavenging activity across all concentrations (up to 89.58% at 1 mg mL^−1^), both the leaf extract and AgNPs showed moderate antioxidant performance. The lower DPPH scavenging activity of AgNPs may be explained by the partial consumption of antioxidant phytochemicals during the reduction in Ag^+^ ions in the biosynthesis process, as well as their involvement in nanoparticle stabilization rather than free radical neutralization. Similar observations have been reported in previous studies on green-synthesized silver nanoparticles, where the antioxidant activity of nanoparticles was found to be lower than that of the corresponding plant extracts [7,56,57].

The ABTS radical scavenging activity of *S. tomentosa* leaf extract, biosynthesized AgNPs, and the reference antioxidant BHA is shown in Figure 10. Similar to the DPPH assay, all samples exhibited antioxidant activity; however, notable differences in scavenging efficiency were observed between the two assays. The leaf extract demonstrated strong ABTS radical scavenging activity, reaching 89.52% inhibition at 1 mg mL^−1^, which is comparable to the activity of BHA (94.91%). This result indicates the high electron-donating capacity of *S. tomentosa* phytochemicals.

In contrast to the DPPH results, the biosynthesized AgNPs exhibited moderate and relatively concentration-independent ABTS scavenging activity, with inhibition values remaining around 46–47% across all tested concentrations. This behavior may be attributed to the different reaction mechanisms of the two assays. While DPPH primarily measures hydrogen atom transfer, ABTS involves both hydrogen atom and single-electron transfer mechanisms, making it more sensitive to surface-bound antioxidants. The stable ABTS scavenging activity observed for AgNPs suggests that phytochemicals adsorbed on the nanoparticle surface contribute to electron transfer reactions, even when their free radical scavenging capacity is limited.

When comparing both assays, *S. tomentosa* leaf extract consistently showed higher antioxidant activity than AgNPs, whereas BHA exhibited the strongest radical scavenging performance in both tests. The reduced antioxidant capacity of AgNPs relative to the extract can be explained by the consumption of phenolic compounds during nanoparticle synthesis and their subsequent involvement in capping and stabilization rather than direct radical neutralization. Similar trends have been reported for other plant-mediated silver nanoparticles, highlighting the influence of synthesis routes and surface chemistry on antioxidant behavior [8,58].

### 3.5. Phenolic Content Analysis

In the present study, the total phenolic content of *S. tomentosa* leaf extract was determined as 206.2 mg GAE/g, while the biosynthesized AgNPs exhibited a slightly lower phenolic content of 164.2 mg GAE/g. The higher phenolic content observed in the leaf extract is consistent with the well-documented richness of *Salvia* species in phenolic acids and flavonoids, which are primarily responsible for their strong antioxidant capacity.

The reduction in total phenolic content following AgNP synthesis can be attributed to the involvement of phenolic compounds in the bioreduction of Ag^+^ ions and their subsequent adsorption onto the nanoparticle surface as capping agents. During the green synthesis process, phenolic hydroxyl groups donate electrons to silver ions, facilitating nanoparticle formation, which may lead to partial consumption or structural modification of these compounds. Consequently, the measured phenolic content of AgNPs is lower than that of the crude plant extract.

Despite this decrease, the relatively high phenolic content retained in AgNPs (164.2 mg GAE/g) indicates successful surface functionalization with bioactive phytochemicals. This surface-bound phenolic layer may contribute to the observed antioxidant activity of AgNPs in DPPH and ABTS assays and enhance their biological compatibility. Similar trends have been reported in previous studies, where biosynthesized AgNPs exhibited lower TPC values than their corresponding plant extracts but maintained significant bioactivity.

Overall, the TPC results support the antioxidant and antimicrobial findings of this study and highlight the dual function of phenolic compounds in both nanoparticle synthesis and bioactivity. The high phenolic content of *S. tomentosa* leaf extract and the substantial retention of phenolics in AgNPs underscore the potential of this plant as an effective green synthesis agent for biologically active silver nanoparticles.

The results of this study collectively reveal a clear relationship between the phytochemical composition of *S. tomentosa* extract, nanoparticle physicochemical characteristics, and their biological interactions. The phenolic-rich nature of the extract facilitates both the reduction in Ag^+^ ions and the formation of a phytochemical capping layer, leading to relatively small, spherical, and surface-functionalized nanoparticles. These surface-bound biomolecules play a critical role in modulating nanoparticle behavior, influencing both colloidal stability and interaction with biological targets. In terms of DNA interaction, the presence of surface-associated phytochemicals and the controlled release of Ag^+^ ions contribute to a non-destructive and reversible binding mode, avoiding strong intercalative interactions while enabling surface association. This behavior reflects the influence of nanoparticle surface chemistry and size on biomolecular interactions. Similarly, the antibacterial activity of the AgNPs is governed by a combination of factors, including nanoparticle size, surface functionalization, and the gradual release of Ag^+^ ions through oxidative dissolution. The phytochemical capping layer may modulate ion release and reduce immediate toxicity, resulting in a more controlled but sustained antibacterial effect compared to AgNO_3_. Taken together, these findings demonstrate that the biosynthesis conditions, particularly the phytochemical profile of the plant extract, directly influence nanoparticle structure, which in turn governs both DNA interaction behavior and antibacterial performance. This integrated relationship highlights the potential of plant-mediated synthesis strategies in tailoring nano-biointerface properties.

## 4. Conclusions

In this study, silver nanoparticles were successfully biosynthesized using *S. tomentosa* leaf extract via an eco-friendly green synthesis approach. The formation of AgNPs was confirmed by a characteristic surface plasmon resonance band at 472 nm in the UV-Vis spectrum. SEM analysis revealed predominantly spherical nanoparticles with particle sizes ranging from 30 to 80 nm, while EDX analysis verified the presence of elemental silver. FTIR results indicated the involvement of plant-derived biomolecules, particularly phenolic compounds, in the reduction and stabilization of AgNPs. XRD analysis confirmed the crystalline nature of the nanoparticles with a face-centered cubic structure.

Biological activity assays demonstrated that the biosynthesized AgNPs exhibited higher antibacterial activity against both Gram-positive (*Bacillus cereus*) and Gram-negative (*Pantoea agglomerans*) bacteria compared to the *S. tomentosa* leaf extract, while AgNO_3_ showed the strongest immediate antibacterial effect due to the rapid availability of free Ag^+^ ions. The enhanced antimicrobial performance of AgNPs is attributed to their nanoscale size, crystalline structure, controlled Ag^+^ release, and surface functionalization with phytochemicals.

DNA binding studies with the combined spectroscopic and electrophoretic findings suggest that AgNPs interact with DNA through a non-intercalative, surface-associated mechanism that preserves overall structural integrity while inducing moderate conformational modulation. The binding constant (Kb ≈ 1.07 × 10^4^ M^−1^) and corresponding ΔG° (~−23 kJ mol^−1^) indicate spontaneous yet reversible affinity, suitable for transient complex formation rather than irreversible stabilization. Despite the pronounced hyperchromism (~220%) reflecting base-stacking perturbation, the absence of EB displacement excludes deep intercalation, while the modest (~10%) decrease in DAPI fluorescence suggests limited groove proximity without strong competitive occupation. Importantly, agarose gel electrophoresis demonstrated preserved plasmid integrity and maintained electrophoretic mobility, confirming that AgNP interaction does not induce extensive strand cleavage or condensation under the tested conditions. Taken together, these results indicate that AgNPs form moderately stable, non-destructive surface complexes with DNA, suggesting their potential for biointerface-related interactions involving nucleic acids. However, further studies, particularly on biocompatibility and toxicity, are required to evaluate their suitability for gene delivery applications.

Antioxidant activity evaluation using DPPH and ABTS assays revealed that the *S. tomentosa* leaf extract possessed strong radical scavenging capacity, which was supported by its high total phenolic content (206.2 mg GAE/g). Although the biosynthesized AgNPs exhibited lower antioxidant activity and total phenolic content (164.2 mg GAE/g) compared to the crude extract, they retained moderate radical scavenging activity, indicating the presence of surface-bound phenolic compounds. The differences observed between DPPH and ABTS assays further highlighted the assay-dependent antioxidant mechanisms of plant-mediated AgNPs.

Overall, *S. tomentosa*-capped AgNPs integrate phytochemical surface functionalization with antibacterial activity and a non-destructive, surface-mediated DNA interaction mode. These features underscore their potential utility as biofunctional nano-biointerfaces, while further work on colloidal stability, cytotoxicity, and performance under physiologically relevant conditions is needed to substantiate application claims.

## Data Availability

The original contributions presented in this study are included in the article. Further inquiries can be directed to the corresponding author.

## References

[1] Karahan H., Çölgeçen H. (2021). The biosynthesis of silver nanoparticles and their use as a biosensor material. Commagene J. Biol..

[2] Takcı D.K., Özdenefe M.S., Genç S. (2023). Green synthesis of silver nanoparticles with antibacterial activity using *Salvia officinalis* aqueous extract. J. Cryst. Growth.

[3] Okaiyeto K., Hoppe H., Okoh A.I. (2021). Plant-based synthesis of silver nanoparticles using aqueous leaf extract of *Salvia officinalis*: Characterization and antiplasmodial activity. J. Clust. Sci..

[4] Rathi B.S., Kumar P.S., Sanjay S., Parthasarathy V., Rangasamy G., Vo D.-V.N. (2025). Innovative eco-friendly silver nanoparticles: Synthesis, characterization and applications. Chem. Eng. Commun..

[5] Ijaz I., Gilani E., Nazir A., Bukhari A. (2020). Detail review on chemical, physical and green synthesis, classification, characterizations and applications of nanoparticles. Green Chem. Lett. Rev..

[6] Duman H., Eker F., Akdaşçi E., Witkowska A.M., Bechelany M., Karav S. (2024). Silver Nanoparticles: A comprehensive review of synthesis methods and chemical and physical properties. Nanomaterials.

[7] Bedlovičová Z., Strapáč I., Baláž M., Salayová A. (2020). A brief overview on antioxidant activity determination of silver nanoparticles. Molecules.

[8] Erenler R., Ojelade R.A., Karan T., Gecer E.N., Genc N., Yaman C. (2023). Facile, efficient synthesis of silver nanoparticles using *Salvia absconditiflora*: Assessment of their antioxidant capacity and catalytic activity. Inorg. Chem. Commun..

[9] Karunakaran G., Sudha K.G., Ali S., Cho E.-B. (2023). Biosynthesis of nanoparticles from various biological sources and its biomedical applications. Molecules.

[10] Ahmed R., Manik K.H., Islam M.S., Rhine A., Mim J.J., Hossain N. (2026). Green synthesis methods for nanoparticles: Principles, biological routes, and physicochemical approaches toward sustainable nanotechnology. Next Mater..

[11] Karahan H.A., Çölgeçen H. (2023). Silver nanoparticles production mediated by natural tetraploid *Trifolium pratense* L.: Characterization and biological activity. Acta Agrobot..

[12] Karahan H., Tetik N., Çölgeçen H. (2023). Phytofabrication of silver nanoparticles using callus extracts of natural tetraploid *Trifolium pratense* L. and its bioactivities. Front. Life Sci. Relat. Technol..

[13] Manzoor S.I., Jabeen F., Patel R., Rizvi M.M.A., Khalid I., Malik M.A., Dar T.A. (2025). Green synthesis of biocompatible silver nanoparticles using *Trillium govanianum* rhizome extract. Mater. Adv..

[14] El Alouani M., Saufi H., Aouan B., Bassam R., Ben Tourtit M., Bassam A., Ahmina W., Rachdi Y., Belaaouad S., Alehyen S. (2026). A comprehensive review on green synthesis and characterization of plant-based nanoparticles for water treatment applications: Adsorption and photodegradation of organic and ınorganic pollutants. Sustainability.

[15] Syed S.M., Kulkarni S., Patil M., Satpute K. (2026). A comprehensive review of green synthesis methods and applications of nanoparticles derived from plant extracts and microorganisms. Discov. Green Chem..

[16] Gecer E.N. (2021). Green synthesis of silver nanoparticles from *Salvia aethiopis* L. and antioxidant activity. J. Inorg. Organomet. Polym. Mater..

[17] Chauhan M., Mori P., Kumar V., Kapadiya K., Masih H., Goswami S. (2025). Harnessing the ethnomedicinal potential of *Cassia fistula*: Biogenic silver nanoparticles for DNA protection and cancer therapy. Microbe.

[18] Bosetti M., Masse A., Tobin E., Cannas M. (2002). Silver-coated materials for fixation devices: Biocompatibility and genotoxicity. Biomaterials.

[19] Panyam J., Labhasetwar V. (2003). Biodegradable nanoparticles for drug and gene delivery to cells and tissue. Adv. Drug Deliv. Rev..

[20] Ginn S.L., Amaya A.K., Alexander I.E., Edelstein M., Abedi M.R. (2018). Gene therapy clinical trials worldwide to 2017: An update. J. Gene Med..

[21] Bhattacharya R., Mukherjee P. (2008). Biological properties of “naked” metal nanoparticles. Adv. Drug Deliv. Rev..

[22] Rai M., Yadav A., Gade A. (2009). Silver nanoparticles as a new generation of antimicrobials. Biotechnol. Adv..

[23] AshaRani P.V., Low Kah Mun G., Hande M.P., Valiyaveettil S. (2009). Cytotoxicity and genotoxicity of silver nanoparticles in human cells. ACS Nano.

[24] Glibitskiy G.M., Jelali V.V., Semenov M.O., Roshal A.D., Glibitskiy D.M., Volyanskiy O.Y., Zegrya G.G. (2012). Interaction of DNA with silver nanoparticles. Ukr. J. Phys..

[25] Zhang X.-F., Liu Z.-G., Shen W., Gurunathan S. (2016). Silver nanoparticles: Synthesis, characterization, properties, applications, and therapeutic approaches. Int. J. Mol. Sci..

[26] Tepe B., Daferera D., Sokmen A., Sokmen M., Polissiou M. (2005). Antimicrobial and antioxidant activities of the essential oil and various extracts of *Salvia tomentosa* Miller (Lamiaceae). Food Chem..

[27] Zeki M., Haznedaroglu N., Karabay N.U., Zeybek U. (2001). Antibacterial activity of *Salvia tomentosa* essential oil. Fitoterapia.

[28] Ulukanli Z., Karabörklü S., Cenet M., Sagdic O., Ozturk I., Balcilar M. (2013). Essential oil composition, insecticidal and antibacterial activities of *Salvia tomentosa* Miller. Med. Chem. Res..

[29] Piątczak E., Kolniak-Ostek J., Gonciarz W., Lisiecki P., Kalinowska-Lis U., Szemraj M., Chmiela M., Zielińska S. (2024). The effect of *Salvia tomentosa* Miller extracts, rich in rosmarinic, salvianolic and lithospermic acids, on bacteria causing opportunistic infections. Molecules.

[30] Kahraman H.A., Usluer M.S., Kaya M.M., Tutun S., Tutun H., Demir M.M., Sevin S. (2022). Antimicrobial potential and phenolic composition of *Salvia tomentosa* extract against some pathogenic bacteria. Fresenius Environ. Bull..

[31] Balkir S., Hazman O., Aksoy L., Yilmaz M.A., Cakir O., Erol I. (2023). Phytochemical profile, antioxidant and antimicrobial potency of aerial parts of *Salvia tomentosa* Miller. Acta Chim. Slov..

[32] Zhumaliyeva G., Zhussupova A., Zhusupova G.E., Błońska-Sikora E., Cerreto A., Omirbekova N., Zhunusbayeva Z., Gemejiyeva N., Ramazanova M., Wrzosek M. (2023). Natural compounds of *Salvia* L. genus and molecular mechanism of their biological activity. Biomedicines.

[33] Quradha M.M., Duru M.E., Kucukaydin S., Tamfu A.N., Iqbal M., Bibi H., Khan R., Ceylan O. (2024). Comparative assessment of phenolic composition profile and biological activities of green extract and conventional extracts of *Salvia sclarea*. Sci. Rep..

[34] Zarei Z., Azarnivand H., Moazeni M., Bahmani M., Razmjoue D., Oroojalian F. (2025). *Salvia sclarea* L. mediated green synthesis of gold nanoparticles (AuNPs) and evaluation of their antibacterial, anticandidal, and scolicidal properties. Sci. Rep..

[35] Geremew A., Gonzalles J., Peace E., Woldesenbet S., Reeves S., Brooks N., Carson L. (2024). Green synthesis of novel silver nanoparticles using *Salvia blepharophylla* and *Salvia greggii*: Antioxidant and antidiabetic potential and effect on food-borne bacterial pathogens. Int. J. Mol. Sci..

[36] Khan M., Khan T., Wahab S., Aasim M., Sherazi T.A., Zahoor M., Yun S.-I. (2023). Solvent based fractional biosynthesis, phytochemical analysis, and biological activity of silver nanoparticles obtained from the extract of *Salvia moorcroftiana*. PLoS ONE.

[37] Pirtarighat S., Ghannadnia M., Baghshahi S. (2019). Green synthesis of silver nanoparticles using the plant extract of *Salvia spinosa* grown in vitro and their antibacterial activity assessment. J. Nanostructure Chem..

[38] Balčiūnaitienė A., Liaudanskas M., Puzerytė V., Viškelis J., Janulis V., Viškelis P., Griškonis E., Jankauskaitė V. (2022). *Eucalyptus globulus* and *Salvia officinalis* extracts mediated green synthesis of silver nanoparticles and their application as an antioxidant and antimicrobial agent. Plants.

[39] Wang C.K., Lee W.H. (1996). Separation, characteristics, and biological activities of phenolics in areca fruit. J. Agric. Food Chem..

[40] Mihailović V., Srećković N., Nedić Z.P., Dimitrijević S., Matić M., Obradović A., Selaković D., Rosić G., Katanic Stan-ković J.S. (2023). Green synthesis of silver nanoparticles using *Salvia verticillata* and *Filipendula ulmaria* extracts: Optimization of synthesis, biological activities, and catalytic properties. Molecules.

[41] Singleton V.L., Orthofer R., Lamuela-Raventós R.M. (1999). Analysis of total phenols and other oxidation substrates and antioxidants by means of Folin–Ciocalteu reagent. Methods Enzymol..

[42] Harris N., Blaber M.G., Schatz G.C., Bhushan B. (2012). Optical properties of metal nanoparticles. Encyclopedia of Nanotechnology.

[43] Wahab S., Asmare M.M., Khan A., Khan T., Ahmad R., Kim S., Yun S. (2025). Green synthesis of silver nanoparticles using *Salvia rosmarinus* extract: Characterization, mechanistic antibacterial, antibiofilm, and in silico evaluation. Ind. Crops Prod..

[44] Fahim M., Shahzai A., Nishat N., Jahan A., Bhat T.A., Inam A. (2024). Green synthesis of silver nanoparticles: A comprehensive review of methods, influencing factors, and applications. J. Colloid Interface Sci. Open.

[45] Ödemiş Ö., Özdemir S., Gonca S., Arslantaş A., Ağirtaş M.S. (2022). The study on biological activities of silver nanoparticles produced via green synthesis method using *Salvia officinalis* and *Thymus vulgaris*. Turk. J. Chem..

[46] Caymaz B., Yıldız U., Akkoç S., Gerçek Z., Şengül A., Coban B. (2020). Synthesis, characterization, and antiproliferative activity studies of novel benzimidazole-imidazopyridine conjugates as DNA groove binders. ChemistrySelect.

[47] Atabey-Özdemir B., Demirkıran O., Yıldız U., Tekin I.O., Coban B. (2017). Cytotoxicity and DNA binding of copper(II) and zinc(II) complexes of flavonoids: Quercitrin, myricitrin, rutin. Bulg. Chem. Commun..

[48] Chandraker S.K., Lal M., Khanam F., Dhruve P., Singh R.P., Shukla R. (2022). Therapeutic potential of biogenic and optimized silver nanoparticles using *Rubia cordifolia* L. leaf extract. Sci. Rep..

[49] Velammal S.P., Devi T.A., Amaladhas T.P. (2016). Antioxidant, antimicrobial and cytotoxic activities of silver and gold nanoparticles synthesized using *Plumbago zeylanica* bark. J. Nanostructure Chem..

[50] Komal, Sonia, Kukreti S., Kaushik M. (2019). Exploring the potential of environmentally friendly silver nanoparticles for DNA interaction: Physicochemical approach. J. Photochem. Photobiol. B.

[51] Dakal T.C., Kumar A., Majumdar R.S., Yadav V. (2016). Mechanistic basis of antimicrobial actions of silver nanoparticles. Front. Microbiol..

[52] Durán N., Duran M., de Jesus M.B., Seabra A.B., Favaro W.J., Nakazato G. (2016). Silver nanoparticles: A new view on mechanistic aspects on antimicrobial activity. Nanomedicine.

[53] Morones J.R., Elechiguerra J.L., Camacho A., Holt K., Kouri J.B., Ramirez J.T., Yacaman M.J. (2005). The bactericidal effect of silver nanoparticles. Nanotechnology.

[54] Girma A., Alamnie G., Bekele T., Mebratie G., Mekuye B., Abera B., Jufar D. (2024). Green-synthesised silver nanoparticles: Antibacterial activity and alternative mechanisms of action to combat multidrug-resistant bacterial pathogens: A systematic literature review. Green Chem. Lett. Rev..

[55] Eker F., Akdasci E., Duman H., Bechelany M., Karav S. (2025). Green synthesis of silver nanoparticles using plant extracts: A comprehensive review of physicochemical properties and multifunctional applications. Int. J. Mol. Sci..

[56] Kora A.J., Rastogi L. (2013). Enhancement of antibacterial activity of capped silver nanoparticles in combination with antibiotics on model Gram-negative and Gram-positive bacteria. Bioinorg. Chem. Appl..

[57] Khorrami S., Zarepour A., Zarrabi A. (2019). Green synthesis of silver nanoparticles at low temperature in a fast pace with unique DPPH radical scavenging and selective cytotoxicity against MCF-7 and BT-20 tumor cell lines. Biotechnol. Rep..

[58] Dėnė L., Chrapačienė S., Laurinaitytė G., Rudinskaitė A., Viškelis J., Viškelis P., Balčiūnaitienė A. (2024). Green synthesis of silver nanoparticles with *Hyssopus officinalis* and *Salvia officinalis* extracts, their properties, and antifungal activity on *Fusarium* spp. Plants.

